# Survey of ENT services in sub-Saharan Africa: little progress between 2009 and 2015

**DOI:** 10.1080/16549716.2017.1289736

**Published:** 2017-05-09

**Authors:** Wakisa Mulwafu, Robbert Ensink, Hannah Kuper, Johannes Fagan

**Affiliations:** ^a^ Department of Surgery, College of Medicine, Blantyre, Malawi; ^b^ Centre for International Health, University of Bergen, Bergen, Norway; ^c^ Division of Oto-rhino-laryngology, Gelre Hospitals Zutphen, The Netherlands; ^d^ International Centre for Evidence in Disability (ICED), London School of Hygiene and Tropical Medicine, London, UK; ^e^ Division of Otolaryngology, University of Cape Town, Cape Town, South Africa

**Keywords:** Ear nose and throat, audiology, speech therapy, sub-Saharan Africa

## Abstract

**Background**: A 2009 survey of ENT, audiology, and speech therapy services and training opportunities in 18 Sub-Saharan African countries reported that the availability of services was extremely poor, the distribution of services was very inequitable, and training opportunities were limited.

​​**Objective**: We conducted a new survey to determine the current status of ear, nose, and throat (ENT), audiology, and speech therapy services in sub-Saharan Africa.

**Method**: This study is a cross-sectional study. A questionnaire was distributed by email to an ad hoc group of ENT surgeons and audiologists in 30 sub-Saharan African countries. Data from the current survey were compared to those of a 2009 survey. The numbers of ENT surgeons, audiologists, and speech therapists/100,000 people were compared to the ratios in the United Kingdom.

**Results**: A total of 22 countries responded to the questionnaire. When data of the 15 countries that responded in both 2009 and 2015 are compared, the number of ENT surgeons had increased by 43%, audiologists had increased by 2.5%, and speech therapists by 30%. When the 23% population growth is taken into account, the numbers of ENT surgeons, audiologists, and speech therapists per 100,000 people had declined in four countries, and there remains a severe shortfall of ENT surgeons, audiologists, and speech therapists when compared to the UK Respondents cited lack of availability of basic equipment as the most frequent limitation in providing ENT services. Other important factors causing limitations in daily practice were: lack of ENT training facilities and audiological rehabilitation, low awareness of the burden of ENT pathology, as well as poor human resources management.

**Conclusions**: There has been a lack of progress in ENT, audiology, and speech therapy services and training opportunities in sub-Saharan Africa between 2009 and 2015. There is a need to look at increased collaboration with developed countries and non-governmental organisations, establishing new and improving existing training centres in Africa, and task-shifting of some ENT services to primary health workers.

## Background

Hearing impairment is more prevalent in sub-Saharan Africa than in other parts of the world [1]. It is likely to become increasingly common over the coming decades as the population continues to age given that hearing impairment is most prevalent in older age groups. Furthermore, the age-specific prevalence of hearing impairment may also increase for a variety of reasons. In the coming decade, HIV and tuberculosis will become more chronic conditions, thanks to the scaling up of antiretroviral therapy (ART)​​ and other treatments, and so the burden of chronic suppurative otitis media (CSOM) and associated hearing loss will likely increase dramatically [2,3]. It is also predicted that 70% of cancers will occur in developing countries by the year 2030, including those relating to ear, nose, and throat (ENT) [4]. As a consequence, there is a great and growing demand for ENT services in sub-Saharan Africa.

A 2009 survey of ENT, audiology, and speech therapy services and training opportunities in 18 sub-Saharan African countries reported that the availability of services was extremely poor, the distribution of services was very inequitable, and training opportunities were limited [5]. As a consequence, people will not be able to access the services that they require and so will have hearing loss that may have been prevented and is now untreated, with consequent negative impacts on quality of life, mental health, and economic productivity. Furthermore, in Malawi, as elsewhere in Africa, the large burden of head and neck cancer is treated largely surgically, in the absence of radiotherapy services, so that a lack of ENT surgeons will increase mortality rates. Overall, the goal to achieve Universal Health Coverage will not be achieved without adequate ENT services [6]. Nor will the Sustainable Development Goal to ‘Ensure healthy lives and promote well-being for all at all ages’, since good hearing is fundamental to health and quality of life.

Since 2009, six new ENT training programmes have been established in sub-Saharan countries, ​​10 head and neck surgeons has completed the University of Cape Town Karl Storz Fellowship in Advanced Head and Neck Surgery, all of whom have returned to teaching hospitals in their own countries, and new audiology and speech therapy training programmes have been established in Ghana and Kenya. The authors therefore thought it timely to repeat the 2009 survey to determine the current status of ENT, audiology, and speech therapy services in sub-Saharan Africa, and to reassess the extent and appropriateness of these services. These data are important to plan and promote effective, targeted support, to initiate regional training initiatives for these services, and to raise awareness about the need to develop ENT, audiology, and speech therapy services in Africa.

## Methods

### Selection of study subjects

An ad hoc group of ENT surgeons and audiologists in 30 out of 48 sub-Saharan African countries in which there were known to be ENT services were traced through personal contacts of three of the authors (WKM, RJHE, JJF). The authors were unable to contact people in the two Portuguese-speaking countries (Mozambique and Angola). Although the questionnaire was translated into French the responses from West African countries were low.

### Procedure

A questionnaire was distributed by email (see the Appendix), with emails obtained through personal contacts. Those who did not respond to the emails were telephoned and reminded about the questionnaire. Email reminders were sent a maximum of 12 times.

### Material

Questions were asked about the availability of ENT, audiology, and speech therapy services and equipment (nil/poor/good/excellent), about training programmes for ENT surgeons, audiologists, and speech therapists, about the availability of services in rural areas, and about their opinions about how to improve the situation. The rating system used for availability of services was as follows:

• Nil: absent services scored;

• Poor: less than half of population has access to care;

• Good: most but not all have access to care;

• Excellent: almost all have access to care.

### Data analysis

The numbers of ENT surgeons, audiologists, and speech therapists/100,000 people in sub-Saharan Africa were compared to the ratios in the United Kingdom (UK) [7]. Data were compared to the 2009 survey. Data analysis was done using SPSS version 21 using descriptive statistics. Categorical variables were represented by frequency and ratios.

## Results

**Figure 1.**
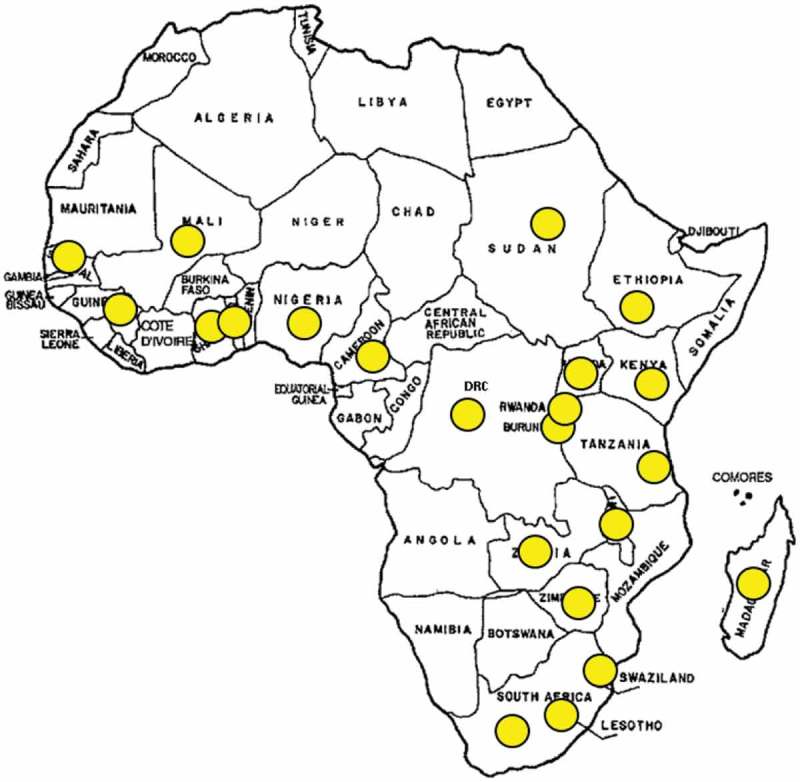
Twenty-two countries that participated in the current study.​​

Twenty ENT surgeons and 2 audiologists from 22 sub-Saharan countries responded to the survey, giving a response rate of 73% (Figure 1). Fifteen countries responded in both 2009 and 2015 and progress in these countries could therefore be compared. Three countries (Botswana, Ivory Coast, and Namibia) that had been surveyed in the 2009 study did not respond and a further seven countries (Burundi, Cameroon, Guinea Conakry, Mali, Rwanda, Sudan, and Togo)​​ responded in 2015 but not in 2009.

The total population of the 22 countries represented in the study was 720,500,000; this represents 75% of the population of sub-Saharan Africa. Among the 22 countries that were sampled, there were a reported total of 847 ENT surgeons, 580 audiologists, 906 speech therapists, 264 ENT clinical officers, and 320 oncologists. When data are pooled across the sample, the regional ratio was 1.2 million people per ENT surgeon, 0.8 million people per audiologist, and 1.3 million people per speech therapist.

When data of the 15 countries that responded in both 2009 and 2015 are compared, the total number of ENT surgeons had increased from 442 to 634, representing a 43% increase (mean increase from 15 to 18 per country), the total number of audiologists had increased from 511 to 524, representing a 2.5% increase (mean increase from 1 to 3 per country), and the total number of speech therapists had increased from 1164 to 1514, representing a 30% increase (median increase from 2 to 3 per country).​​ The number of audiologists and speech therapists had increased in 86% of the countries, although the actual numbers of audiologists and speech therapists are extremely low if South Africa, Kenya, and Sudan are excluded (Table 1).

**Table 1.** Comparison of total numbers of ENT surgeons, audiologists, and speech therapists per country in 2009 and 2015 (* no data for 2009).​​

	ENT surgeons		Audiology		Speech therapy	
	2009	2015	2009	2015	2009	2015
Burundi	*	6	*	10	*	0
Cameroon	*	35	*	0	*	25
D.R.C	25	18	0	3	0	3
Ethiopia	11	22	0	1	0	1
Ghana	15	27	6	13	2	6
Guinea Conakry	*	6	*	6	*	1
Kenya	40	76	4	7	3	16
Lesotho	2	2	0	2	0	1
Madagascar	16	15	2	10	4	1
Malawi	1	2	0	3	0	0
Mali	*	15	*	0	*	2
Nigeria	70	140	5	13	3	4
Rwanda	*	8	*	4	*	1
S. Africa	200	246	490	444	1144	1470
Senegal	25	15	1	3	2	4
Sudan	*	105	*	5	*	2
Swaziland	2	3	1	5	1	3
Tanzania	11	18	0	1	2	3
Togo	*	8	*	0	*	25
Uganda	16	35	1	15	2	0
Zambia	2	7	1	1	0	1
Zimbabwe	6	8	0	3	1	1

However, there had been a large population growth (23%) in the countries surveyed between 2009 and 2015, from 486 million to 599 million people. When this population growth is taken into account when calculating the numbers of ENT surgeons, audiologists, and speech therapists per 100,000 people, it was found that in four countries (D.R.C, Lesotho, Madagascar, Senegal) there had been a decline in the number of ENT surgeons per 100,000 people between 2009 and 2015, while in Ghana, Kenya, and Zambia there had been an improvement in the numbers of ENTs, audiologists, and speech therapists per 100,000 people (Table 2). There remains a severe shortfall of ENT surgeons, audiologists, and speech therapists across all countries when compared to the UK (Table 2).

**Table 2.** ENT surgeons, audiologists, and speech therapists/100,000 people in 2009 and 2015 compared to the UK

	ENT surgeons		Audiology		Speech therapy	
	2009	2015	2009	2015	2009	2015
Burundi	*	0.056	*	0.093	*	0.000
Cameroon	*	0.150	*	–	*	0.107
D.R.C	0.045	0.025	–	0.004	–	0.004
Ethiopia	0.014	0.022	–	0.001	–	0.001
Ghana	0.068	0.104	0.022	0.048	0.007	0.022
Guinea	*	0.049	*	0.049	*	0.008
Kenya	0.121	0.163	0.012	0.015	0.009	0.034
Lesotho	0.1	0.094	–	0.047	–	0.047
Madagascar	0.09	0.062	0.012	0.041	0.024	0.004
Malawi	0.01	0.012	–	0.017	–	0.000
Mali	*	0.092	*	–	*	0.011
Nigeria	0.054	0.076	0.004	0.007	0.002	0.002
Rwanda	*	0.064	*	0.032	*	0.008
S. Africa	0.417	0.46	1.021	0.827	2.383	2.748
Senegal	0.227	0.100	0.009	0.021	0.018	0.029
Sudan	*	0.265	*	0.013	*	0.007
Swaziland	0.2	0.233	0.1	0.039	0.1	0.233
Tanzania	0.031	0.034	–	0.002	0.006	0.006
Togo	*	0.111	*	–	*	0.417
Uganda	0.057	0.087	0.004	0.037	0.007	0.016
Zambia	0.017	0.045	0.004	0.006	–	0.006
Zimbabwe	0.043	0.053	–	0.020	0.007	0.007
UK	1.0	2.36	4.1		16.393	

Note: Shaded cells indicate declining numbers/100,000 people; no data for 2009 denoted by * [5]. DRC: Democratic Republic of the Congo; U.K: United Kingdom.​​​​

New training programmes had been introduced in six countries since the 2009 study (Table 3). In three countries (Rwanda, Zimbabwe, and Ethiopia) new ENT training programmes represent the only training programmes in the respective countries. One new audiology (Ghana) and one speech therapy (Kenya) programme had been introduced in the time between the two studies. However, there has been little overall change in the numbers of new ENT surgeons, audiologists, and speech therapists qualifying per annum. Five countries (Malawi, Kenya, Mali, Togo, and Cameroon) reported to have training programmes for ENT clinical officers (non-doctors who undergo an 18-month training programme in basic ENT diagnostic and therapeutic skills such as removing foreign bodies, and performing tonsillectomies and adenoidectomies).

**Table 3.** Training programmes.

Countries	Number of medical schools		ENT training				Audiology training programmes		Speech training programmes	
			Training programmes		New ENTs p.a					
	2009	2015	2009	2015	2009	2015	2009	2015	2009	2015
Burundi	*	3	*	1	*	1	*	–	*	–
Cameroon	*	4	*	1	*	5	*	–	*	–
D.R.C	4	6	1	1	1	2	–	–	–	–
Ethiopia	6	9	–	1	–	4	–	–	–	–
Ghana	3	4	2	2	1–2	2	–	Yes	–	–
Guinea Conakry	*	3	*	3	*	5	*	–	*	–
Kenya	2	6	1	1	4	1	Yes	Yes	–	Yes
Lesotho	–	1	–	–	–	–	–	–	–	–
Madagascar	2	6	1	1	0	1	–	–	–	–
Malawi	1	1	0	1	0	0	–	–	–	–
Mali	*	3	*	1	*	4	*	–	*	–
Nigeria	36	58	19	37	4	5	–	–	–	–
Rwanda	*	2	*	1	*	2	*	–	​–​	–
S. Africa	8	9	8	9	6	6	Yes	Yes	Yes	Yes
Senegal	2	5	1	1	5	4	–	–	–	–
Sudan	*	95	*	0	*	10	*	–	​–​	–
Swaziland	–	–	–	–	–	–	–	–	–	–
Tanzania	5	4	2	2	2	3	–	–	–	–
Togo	*	1	*	1	*	?	*	–	*	Yes
Uganda	3	3	1	2	1–3	4	–	–	–	–
Zambia	1	3	–	–	–	–	–	–	–	–
Zimbabwe	1	1	–	1	–	2	–	–	–	–

Note: Shaded cells indicate new training programmes established since the 2009 study.

Table 4 illustrates the poor state of ENT, audiology, and speech therapy services in state hospitals in the 22 African countries polled. Only three countries (Malawi, Burundi, and Ethiopia) provide ENT services for free in state hospitals. Sinus and rhinologic surgery had 66% ‘poor’ or ‘nil’ availability. Audiology and otologic surgery had 87% ‘nil’ or ‘poor’ availability. Head and neck oncologic surgery had 75% ‘nil’ or ‘poor’ availability. A big need in ENT practice appears to be equipment for otologic surgery and basic equipment. The availability of modern medical equipment remains problematic with 68% reporting ‘nil’ or ‘poor’ availability.

**Table 4.** Numbers of countries with nil/poor/good/excellent services in state hospitals.

	Availability in state service			
	Nil	Poor	Good	Excellent
**Sinus and rhinology surgery**				
Endoscopic ethmoid sinus surgery	8	10	3	1
External ethmoidectomy	4	5	9	3
Inferior meatal antrostomy	4	10	4	3
Caldwell Luc/radical antrostomy	4	4	9	4
Cosmetic rhinoplasty	12	9	0	1
				
**Audiology and otologic surgery**				
Audiology	0	15	5	1
Auditory brainstem reflexes (ABR)	9	12	1	0
Otoacoustic emissions (OAE) screening​​	7	13	2	0
Hearing screening: newborn	18	3	1	0
Hearing screening: schools	11	11	0	0
Hearing screening: industry	9	13	0	0
Hearing aids	5	14	3	0
Myringotomies, ventilation tubes	0	14	6	2
Tympanoplasty	2	13	5	2
Mastoidectomy for cholesteatoma	0	14	5	3
Mastoidectomy for mastoiditis	1	13	5	0
Middle ear (ossicular) prostheses	14	7	1	0
Bone anchored hearing aids	19	3	0	0
Cochlear implants	18	4	0	0
				
**Head and neck oncologic surgery**				
Total laryngectomy	6	7	5	4
Speech prosthesis post-laryngectomy	16	4	1	1
Partial laryngectomy	12	6	3	1
CO_2_ laser surgery	19	3	0	0
Parotidectomy	1	5	11	4
Radical neck dissection	6	9	3	4
Modified neck dissection	7	8	3	4
Selective neck dissection	7	9	2	4
Commando resection	11	7	3	1
Total maxillectomy	5	10	3	4
Craniofacial resection	10	10	1	1
Pedicled flaps e.g. pectoralis major	8	7	4	3
Free microvascular flaps	13	5	3	0
Mini and microplates	15	5	2	0
Fine needle aspiration	2	8	7	6
Frozen section	15	5	2	0
				
**High-cost equipment and services**				
Flexible nasopharyngoscopy (NR1)	2	12	3	5
Operating microscopes	1	14	4	3
Otology drill	2	14	4	2
CO_2_ laser	17	3	1	0
Ultrasound of neck	2	4	11	5
Computerized tomography (CT) scanning​​	1	9	9	3
Magnetic resonance imaging (MRI) scanning	7	9	4	2
Positron emission tomography (PET) scanning	20	2	0	0
Radiation therapy	8	7	7	0

Twenty of the 22 countries polled had schools for the deaf; the median number of deaf schools per country was 6 (range 1–120).

Availability of services outside major cities was shown to be a problem in 2009, and remains a concern in 2015. In 2015, all the respondents reported that availability of ENT services outside of the capital city was ‘nil’ (n = 8) or ‘poor’ (n = 8). In 2009, the availability of services outside the capital was mostly reported as ‘nil’ (n = 9) or ‘poor’ (n = 3), with some countries reporting it as being ‘good’ (n = 5) or ‘excellent’ (n = 1).

Respondents cited lack of availability of basic equipment as the most frequent limitation in providing ENT services, but they also cited poor ENT training facilities, audiological rehabilitation, and awareness of the burden of ENT pathology, as well as human resources management as among the top limitations encountered in daily practice.

## Discussion

This study reports the current state of ENT, audiology, and speech therapy services and training opportunities in sub-Saharan Africa and compares it to a previous study undertaken in 2009. It is clear from the results that there has been little progress since 2009.

Although the absolute numbers of ENT surgeons, audiologists, and speech therapists have increased, the ratios to the populations in the individual countries have increased only marginally in some countries while in others they have declined due to rapid population growth. Comparing these ratios to the UK, sub-Saharan Africa has extremely low coverage of ENT, audiology, and speech therapy services. This trend has also been observed in eye health where the regional practitioner ratio of 2.9 per million people for sub-Saharan Africa was way below the Vision 2020 target of 4 per million people; that study called for substantial and more targeted investment in human resources for eye health if Vision 2020 aims for the prevention of avoidable blindness were to be achieved for sub-Saharan Africa [8].

The availability of equipment remains poor with most (66–87%) countries rating the availability of equipment between ‘nil’ or ‘poor’. Poor infrastructure and equipment are a deterrent to working in such countries. There is clearly a need to invest in infrastructure and equipment and to create training centres for ENT specialists, audiologists, and speech therapists. Training within Africa will also make it more likely that graduates remain and work in Africa.

The number of ENT, audiology, and speech therapy training programmes has stagnated. Any increases in training programmes and numbers of graduates have been offset by the large (23%) population increase in the countries surveyed. It is possible to increase the number of training programmes and number of ENT surgeons, audiologists, and speech therapists qualifying in Africa, but this will require deliberate investments in such training, staffing, and infrastructure. It is unlikely that sub-Saharan Africa can meet this training obligation alone in the short to medium term. It requires assistance from high-income countries. This can be achieved partly by collaborative programmes with countries or organisations in high-income settings. There have been examples of such collaborative programmes that have helped train ENT cadres in Africa [9–12]. For instance, CBM International has helped build ENT units in Zambia, Malawi, and Zimbabwe. The University of Cape Town Karl Storz Head and Neck Fellowship has trained 10 Head and Neck Fellows in Africa. Operation Ear Drop Kenya in collaboration with the University of Nairobi have fully equipped a permanent temporal lab at the University of Nairobi and have conducted temporal bone courses every year since 1987. Improved regional collaboration through the College of Surgeons of East, Central and Southern Africa (COSECSA) needs to be fostered to permit training in smaller units. Increasing training programmes will improve ENT human resources only after a lead time of several years, and so establishing these programmes should not be delayed any further.

The training of primary- and middle-level health workers (‘bottom-up procedure’) can also have an impact on the management of ear and hearing disorders in sub-Saharan Africa, for instance through the management of otitis media. A study on the burden of disease caused by otitis media demonstrates clearly the enormous impact of acute otitis media (AOM)/CSOM​​ on hearing in the African continent [13]. This article stresses that the African continent needs action on ​​otitis media with effusion (OME)/CSOM and its effect on hearing. The high percentages of otitis media found in common daily African ENT practice of 45% of OME in children > 15 yrs and at about 11% of CSOM in adults emphasise this as well. Small changes in the treatment of CSOM were described by Guntinas-Lichius on optimising the pre-treatment process for CSOM in Ethiopia, such as regular cleaning, suctioning, dry mopping under microscopic control, and topical treatment with antibiotic ear drops [14]. Only three countries are training ENT clinical officers. There is need to clearly define what these primary- and middle-level workers are able to do, to develop protocols for task-shifting of activities to these health workers and thereby increase access to ENT services for people outside the cities.

In the current study, the respondents cited lack of availability of basic equipment as the most frequent limitation in providing ENT services. Other key limitations encountered in daily practice included poor ENT training facilities, audiological rehabilitation, and awareness of the burden of ENT pathology in the medical field as well as human resources management. Moving forward, further research is needed to explore which interventions will work to speed up the progress of ENT services in Africa, including developing innovative methods to fill these gaps.

The study has some limitations. We were not able to collect information from all the 48 countries in sub-Saharan Africa. Most countries in sub-Saharan Africa do not keep databases of their health workers and so most of the information was collected from proxy contacts in the respective countries. Another limitation of the study is that we collected the data for the total number of practitioners in the different countries, both active and inactive. Furthermore, the time period between the two surveys may be relatively short for a change to be detected, especially for training programmes of long duration such as those for training ENT surgeons. The major strength of this study, however, is that it provides a database for ENT services in sub-Saharan Africa, a region where data are scarce.

## Conclusions

With little progress in the development of ENT services in Africa, there is need to look at ways of dealing with the increased burden of ENT conditions in the region in order to prevent unnecessary hearing loss and maximise the quality of life of those with untreatable ear conditions, as well as other diseases of the head and neck including cancers. Increased collaboration with high-income countries and non-governmental organisations is required to establish new and to improve existing training centres in Africa, and task-shifting is required of some ENT services to primary- and middle-level health workers.

## Recommendations

- Human resource development by establishing new and improving existing training centres in sub-Saharan Africa.
- Targeted infrastructure development for ENT services in sub-Saharan Africa.
- Monitoring status of ENT conditions and services.
- Increased collaboration with high-income countries and non-governmental organisations in ENT capacity development.
- Task-shifting of some ENT services to primary- and middle-level health workers.

## Paper context

A 2009 survey of ENT, audiology, and speech therapy services and training opportunities in 18 sub-Saharan African countries reported that the availability of services was extremely poor. We report that little progress has been made since 2009. There is an urgent need to increase collaboration with high-income countries and non-governmental organisations to establish new and to improve existing training centres in Africa and for task-shifting of some ENT services to primary- and middle-level health workers.

## Appendix.

**Table:** SURVEY OF EXISTING ENT SERVICES IN AFRICA***Thank you for taking time to fill in this form.***Country

What is the latest population of your country?			
How many of the following do you have in your country	Number/ Comment		
General ENT Surgeons			
Audiologists			
Speech Therapists			
ENT Clinical Officers			
Schools for the deaf			
Training programme			
How many medical schools do you have in your country?			
How many have ENT training programmes? What are they?			
How many ENT surgeons qualify per year?			
Do you have Audiology training programme? If yes, how many people qualify per year?			
Do you have Speech Therapy training programme? If yes, how many people qualify per year?			
Do you have ENT Clinical Officer training programme? How many people qualify per year?			

Guideline for rating services:

NIL = absent;

POOR = Fewer than half who need the service receive it when needed;

GOOD = Most but not all of those that need the service receive it when needed;

EXCELLENT = Virtually all of those that need the service receive it when needed.

Please take note that availability of service/equipment is not mere physical presence of it. For example if C.T Scanning is available but not working, it should be rated as POOR

**Table:**

Please rate the availability of the following services:	Nil	Poor	Good	Excellent
**Hearing-related services in public hospital**				
Audiology				
Auditory Brain Stem Reflexes				
Oto-acoustic emissions				
National newborn screening programme				
Newborn screening programme hospital based				
Hearing screening: schools				
Hearing screening: industries				
Hearing aids				
Myringotomies, Ventilation tubes				
Tympanoplasty				
Mastoidectomy for cholesteatoma				
Mastoidectomy for mastoiditis				
Middle ear (ossicular) prosthesis				
Bone anchored hearing aids				
Cochlear implants				
				
				
**Modern ear equipment in public hospitals**				
Operating microscopes				
Otology drill				
C.A.T scanning				
M.R.I scanning				
				
**Please rate the availability of State ENT services outside major cities**				

**OPEN QUESTIONS**

I: Are Ear services in public hospitals free or are they paid by the patient?

J: What are the biggest needs in general ENT practice in your country?

K: Which limitations occur most frequently in your practice?

L: Please estimate the percentage of Ear services concentrated in your capital city.
